# Immunocytochemical studies on the distribution of arabinogalactan proteins (AGPs) as a response to fungal infection in *Malu*s x *domestica* fruit

**DOI:** 10.1038/s41598-019-54022-3

**Published:** 2019-11-22

**Authors:** Agata Leszczuk, Piotr M. Pieczywek, Agata Gryta, Magdalena Frąc, Artur Zdunek

**Affiliations:** Institute of Agrophysics, Polish Academy of Sciences, Doświadczalna 4, 20-290 Lublin, Poland

**Keywords:** Cell wall, Immunohistochemistry

## Abstract

Arabinogalactan proteins (AGPs) are cell components implicated in plant-microbe interactions. Despite the significance of AGPs in response to stress factors, their distribution during development of fungal disease in fruit is unknown. In our work, *in situ* analysis of AGP arrangement in fruit inoculated with *Penicillium spinulosum* during the consecutive days of infection development was carried out. For immunolocalization of AGPs, samples were incubated with JIM13, MAC207, LM2, and LM14 antibodies recognizing the AGP carbohydrate moieties. To analyse cell walls without proper action of AGP, an experiment with β-glucosyl Yariv reagent specifically binding AGPs was performed. The results showed an increase of signal fluorescence in the fruit after 16 days of fungal disease. Higher amounts of the examined epitopes were observed in the infection-altered sites of the fruit, in close vicinity to a surface filled by fungal spores. The results indicate that the Yariv reagent treatment induced progress of the fungal disease. Changes in the AGP presence during the fungal disease confirmed their involvement in defence against pathogen attack in fruit.

## Introduction

Plants evolve adaptations and defence reactions as a response to biotic and abiotic stress factors. One of the basic strategies against plant pathogens is the remodelling of the cell wall, or more precisely distribution and accumulation of cell wall-associated proteins^1–3^. Hydroxyproline-rich glycoproteins (HRGPs) are involved in cross-linking and strengthening of the cell wall, which provides disease resistance, such as protection against spatial ramification of the pathogen^4–6^. Arabinogalactan proteins (AGPs), i.e. members of the superfamily of structural proteins, are implicated in fundamental processes in plant-microbe interactions^7–9^. These proteoglycans are attached to the plasma membrane by a glycosylphosphatidylinositol (GPI) anchor at their C-terminus domain, and are released to the extracellular matrix through the action of phospholipases (C or D). Cleavage of GPI-anchored AGPs is important for plasma membrane-cell wall interactions and thus AGPs are proposed to play a role in cellular signalling^10^. Furthermore, AGPs are the base of a supra-molecular network called the APAP1 complex, where they act as cross-linkers between cell wall polysaccharides. The well-defined linkages between AGP, pectins, and arabinoxylan indicate their contribution to the establishment of cell wall integrity, whose disruption has effects on whole cell biology^11,12^. The involvement of AGPs in the relationship between plants and pathogens has been mainly studied in roots, where the occurrence of AGP-rich rhizodeposits improve plant condition and crop production. Their connections with xylogalacturonan in biofilm secreted by infected roots provide anchorage for lignification and form a mechanical barrier impervious to fungal hyphae^13^. Cannesan and co-workers^14^ reported that the surfaces of root border and border-like cells contain accessible AGPs participating in the control of early infection of roots by recognition of the pathogen, mediation of the stress signalling response, and secretion to the rhizosphere. Also in banana roots, AGPs are up-regulated by *Fusarium oxysporum* attack on wounded plants, which is correlated with their contribution in plant susceptibility to the pathogen as signalling constituents enhancing cell-cell communication^15^.

In case of fruit, comprehensive studies have been undertaken to elucidate the mechanism of postharvest host-pathogen interactions. The up- or down regulation of the synthesis of cell wall components and cooperation between them are important for fruit metabolism as well as pathogen susceptibility. The infection mechanism induces the activation of cell wall-degrading enzymes, allowing e.g. depolymerisation of pectins^16^ and reduction of the molecular mass of hemicelluloses^17^. The cell wall disassembly activates a novel defence pathway and influences the course of infection by changing the accessibility of substrates to pathogen enzymes^18,19^. Nevertheless, none of these reports addressed the role of AGPs in fruit during fungal attack. In our previous studies, a correlation between the occurrence of specific AGP epitopes and the stage of fruit ripening and senescence as a result of postharvest storage was reported. Our data concerned spatio-temporal-dependent AGP localization in apple fruit tissues, and allow us to propose that AGPs are good candidates for cell wall-plasma membrane anchors throughout fruit maturation^20^. The immunochemical approach used to examine changes in the fruit cell wall at the cellular level showed that the examined proteoglycans occurred in different zones depending on alteration in the cell wall-plasma membrane^21^. Taking into account our previous results and the significant contribution of AGPs to root-pathogen interactions, in the current study, we considered the presumed response of AGPs to fungal disease in fruit. To analyse the regulation of the cellular arrangement of AGP epitopes in relation to fungal infection, immuno-histochemical techniques with well-defined antibodies were used. Here, we present a detailed description of the inconsistent distribution of AGPs after *Penicillium spinulosum* attack during development of the fruit disease. *Penicillium* species are widely reported postharvest pathogens. As well known, *P. spinulosum* is potentially toxic fungi found on plant-origin food products^22–24^. Secondly, the most characteristic criterion in recognition of AGPs is the specific interaction with an AGP-disrupting agent, i.e. the β-glucosyl Yariv reagent (β-GlcY). The target structures for the β-GlcY are β-1,3-galactooligosaccharides, which are conserved carbohydrate moieties of AGPs^25^. Therefore, to elucidate the AGP impact on infected fruit tissue, an experiment with the β-GlcY was performed. The selective binding to AGPs causes perturbation of their biological functions and gives a possibility of wide application of the β-GlcY as a tool to identify the roles of AGPs in plant physiology.

## Methods

Fruits of *Malu*s x *domestica* Borkh. cv. ‘Gala’ were provided by a local producer (Lublin, Poland). Apples were selected based on similar size and at the same stage of ripening; they were harvested at the optimum harvest window for this cultivar (6th Oct. 2018). Apple fruits with similar features and without visible symptoms of the disease and bruising were chosen.

### Preparation of pathogen inoculum

**Figure 1:**
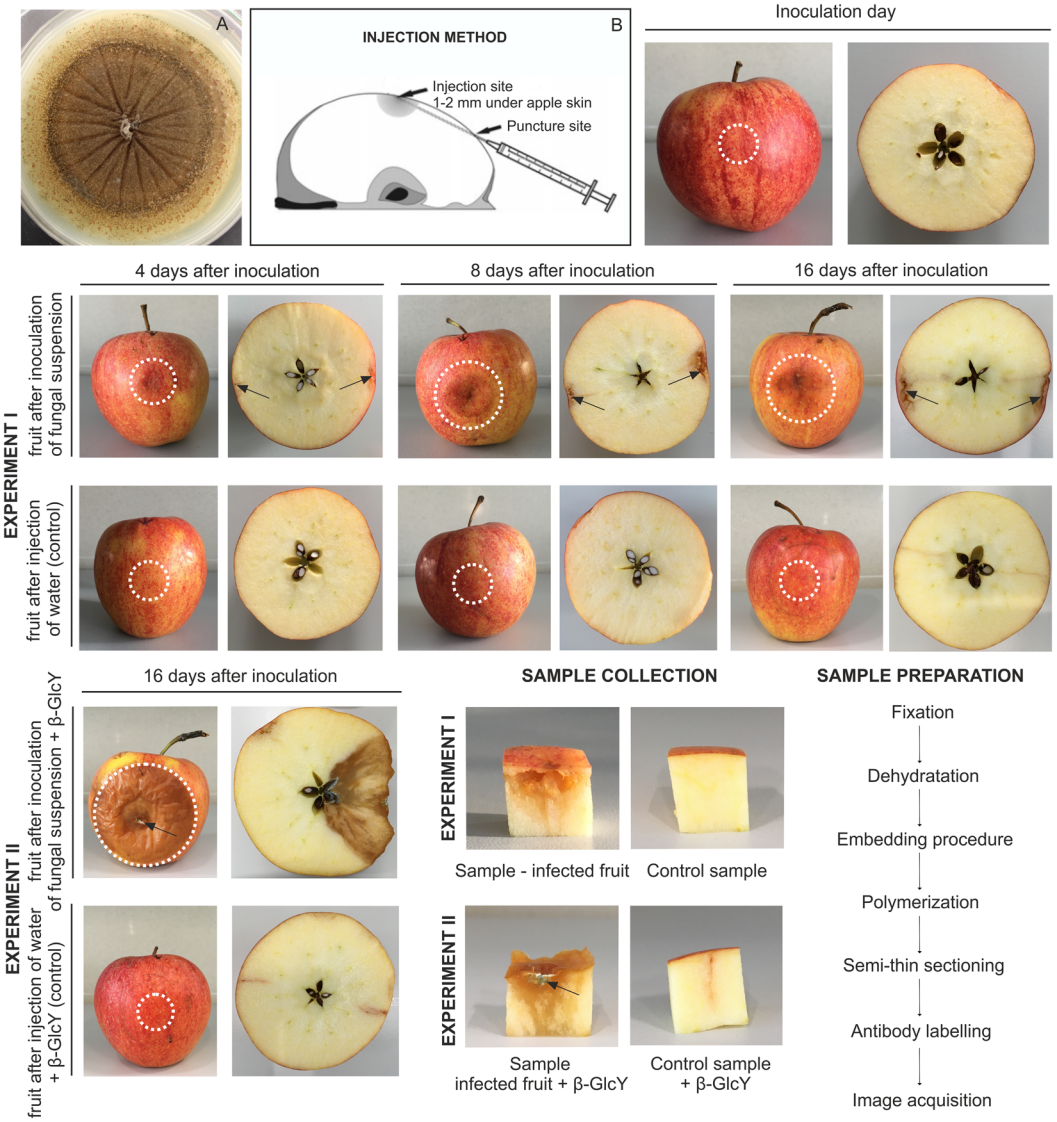
Steps of experiment I and II. Sample collection and sample preparation. 7-day-old culture of *P. spinulosum* on PDA medium (**A**). Injection method reproduced and modified with permission from Kurenda and co-workers^39^ (**B**). Representative images of apple fruits with disease symptoms at different time points (0, 4, 8, and 16 days) after *P. spinulosum* inoculation and corresponding control samples (experiment I) and apple fruit on day 16 after injection with addition of the β-GlcY (experiment II). Arrows and circles indicate sites of injection into the apple fruits.

The pathogen was obtained from the Laboratory of Molecular and Environmental Microbiology, Institute of Agrophysics, Polish Academy of Sciences. *P. spinulosum* strain G259/18 was isolated from infected strawberry plants. In order to isolate pathogenic fungi from diseased strawberry plants, infected parts of plants with visible paralysis: necrotic changes, curled and dried leaves, and bronzed petioles, were selected and washed. Fragments of the plants were rinsed several times in tap water and distilled water; next, they were surface-disinfected with 70% ethanol. The fragments of plant tissue were cut in sterile conditions into small pieces (about 0.5 cm) and placed in selective microbiological medium (Potato Dextrose Agar – PDA, Biocorp, Poland). The PDA plates with the plant parts were incubated in the dark at 23 °C for 5 days. The grown fungi were purified by transferring to fresh PDA medium (Fig. 1A). The fungal sequence was deposited in the GenBank of NCBI (http://www.ncbi.nlm.nih.gov) under the MK801768 accession number.

For inoculation of the fruits, spores from 7-day-old fungal culture were suspended in sterile Milli-Q water. The spores suspension was carefully centrifuged and the final spore concentration was counted under a light microscope using a Thom’s spore counting chamber. The designated fungal spore density was 7 × 10^5^/mL.

### Experiment I - inoculation of apple fruits with the pathogen

0.1 mL of the spores suspension containing 7 × 10^5^ spores per mL was injected into two opposite sides of the apple tissue with a syringe (Fig. 1B). As a control, some fruits were inoculated with 0.1 mL sterile Milli-Q water.

### Experiment II - β-glucosyl Yariv reagent (β-GlcY) treatment

To determine the effect of the perturbed AGP action, the apple fruits were treated with the β-GlcY (1,3,5-tris-(4-β-D-glycopyranosyloxyphenylazo)-2,4,5-trihydroxybenzene). The β-GlcY selectively binds to AGPs, recognizing β-1,3-galactan chains longer than five residues^25^. The 0.1 mL of 30 µM β-GlcY solution and 0.1 mL of the spores suspension were injected into the apple fruits. As a control, some fruits were inoculated with sterile Milli-Q water with addition of a β-GlcY solution. The solution of β-GlcY was prepared as described in the Biosupplies (Australia) protocol.

For both experiments, the fruits were conditioned in normal atmosphere at room temperature for 4, 8, and 16 days. The control fruit were stored in the same conditions as the infected ones. To carry out microscopic analyses, 3 apple fruits for each treatment and time of storage were analysed. After the appropriate incubation time, cube-shaped samples were obtained by excision of the external part of the fruit, which included the epidermal and hypodermal layers, and parenchyma from a depth of ca. 1 cm under the skin. The experiment steps mentioned above are presented in detail in Fig. 1.

### Sample preparation - fixation, resin embedding, and sectioning

For microscopic analysis, the fruit samples were fixed, embedded, and sectioned according to the method described in our previous paper^26^. Briefly, the samples were fixed overnight at room temperature in 2% (w/v) paraformaldehyde and 2.5% (v/v) glutaraldehyde in phosphate buffered saline (PBS, Sigma Aldrich, Saint Louis, USA) and then washed three times in PBS. After dehydratation in a graded series of ethanol solutions from 30% to 96%, the ethanol solution was substituted with dilution series of LR White resin (Sigma Aldrich) and then maintained in 100% resin at 4 °C for 48 h with 3 changes of the resin. The resin-embedding procedure was used following the instructions of the supplier. The samples were placed in gelatin capsules and polymerized at 55 °C for 48 h. Semi-thin sections (1 µm) were obtained using an ultramicrotome (Leica, Austria) with a glass knife and were mounted on poly-L-lysine coated glass slides (Sigma Aldrich).

### Histological staining - toluidine blue, aniline blue

After washing in Milli-Q water, the sections were stained with a 0.5% (w/v) Toluidine blue (Sigma Aldrich) aqueous solution at 55 °C for 30 s in order to examine the anatomical changes in the fruit tissue after the fungal infection. To detect callose fluorescence, the sections were stained with a 0.1% (w/v) Aniline blue (Sigma Aldrich) aqueous solution for 20 min in darkness.

### Indirect immunofluorescence detection of AGPs

A method described in our previous study was used for immunolabelling procedures^26^. At the onset, the collected sections mounted on microscope slides were circled with a liquid blocker (Daido Sangyo, Japan). Slides prepared for immunochemistry reactions were pre-incubated with 1% bovine serum albumin (BSA, Sigma Aldrich) in PBS for 30 min to avoid nonspecific binding of antibodies. The rat IgM primary anti-AGP mAbs JIM13, MAC207, LM2, and LM14 were diluted 1:50 in 0.1% BSA. Fluorescence-labelling was based on the use of goat anti-rat IgM conjugated with AlexaFluor 488 (Cat. No. A21212; ThermoFisher Scientific, USA) adjusted to a 1:200 dilution in 0.1% BSA. After incubation with primary and secondary antibodies, the sections were washed in PBS twice and finally enclosed in Dako Fluorescent Mounting Medium (Sigma Aldrich). For better visualization of the examined epitopes, the sections were counterstained with (0.001%, w/v) Calcofluor White Stain (Sigma Aldrich). Immunofluorescence labelling was carried out on at least several serial sections from each sample of the apple fruit and each kind of antibodies. Control reactions were carried out by incubation in PBS instead of the primary antibody.

### Primary monoclonal antibodies

The epitope of JIM13 is determined to have the structure β-GlcA-(1 → 3)-α-GalA-(1 → 2)-α-Rha units of the carbohydrate motif of AGPs^27,28^. In most studies, MAC207 epitope is reported as the acidic trisaccharide β-GlcA-(1 → 3)-α-GalA-(1 → 2)-α-Rha^28^. LM2 recognises (1 → 6)-β-Gal units with terminal β-GlcA in AGP, β-GlcA-(1 → 6)-β-Gal-(1 → 6)-β-Gal-(1 → 6)-β-Gal-(1 → ^28,29^. LM14 binds to arabinose- and galactose-enriched carbohydrate chains^30^. The antibodies used were obtained from Plant Probes (University of Leeds, UK) and The Complex Carbohydrate Research Center (University of Georgia, USA).

### Fluorescence quantification

All observations were carried out and photographs were taken using a confocal laser scanning microscope (CLSM) Olympus BX51 equipped with corresponding software FluoView v. 5.0. (Olympus Corporation, Tokyo, Japan). Representative image sets were selected and edited using the CorelDrawX6 graphics program.

Optical signal density was calculated as the ratio of the total sum of values of pixels from the green channel (fluorescence indicating the presence of the antibody) within the selected region of interest to the pixel area of this region. The regions of interest had rectangular shapes and were selected manually to cover the tissue area below the epidermal layer of cells. CLSM data was analysed using a script written in Matlab R2010a (MathWorks, USA). The data were statistically analyzed using OriginPro 8.5 software (Origin Lab v8.5 Pro, Northampton, USA). For comparisons of the mean values, an analysis of variance (one-way ANOVA) followed by post hoc Tukey’s honestly significant difference test was used. For all analyses, the significance level was estimated at *p* < 0.05.

## Results

### Morphological changes in fruit tissue after fungal infection

Representative images of morphological changes in the apple fruits are presented in Fig. 1. The first signs of fungal infection were visible as slightly discoloured spots on the fruit tissue on day 4 after the inoculation. However, the most significant changes were noticeable on the 16th day of experiment I. The fruit tissue was remarkably disturbed with light brown watery spots at the inoculation sites. There were no morphological changes in the corresponding control samples injected with water.

In case of experiment II, the addition of the β-GlcY to the inoculum suspension resulted in rapid progress of fungal infection. After 16 days of the experiment, the infected parts of the fruits were completely decayed, with blue-green points typical for blue mould (arrow).

### Histological observations of fruit tissue after fungal infection

**Figure 2:**
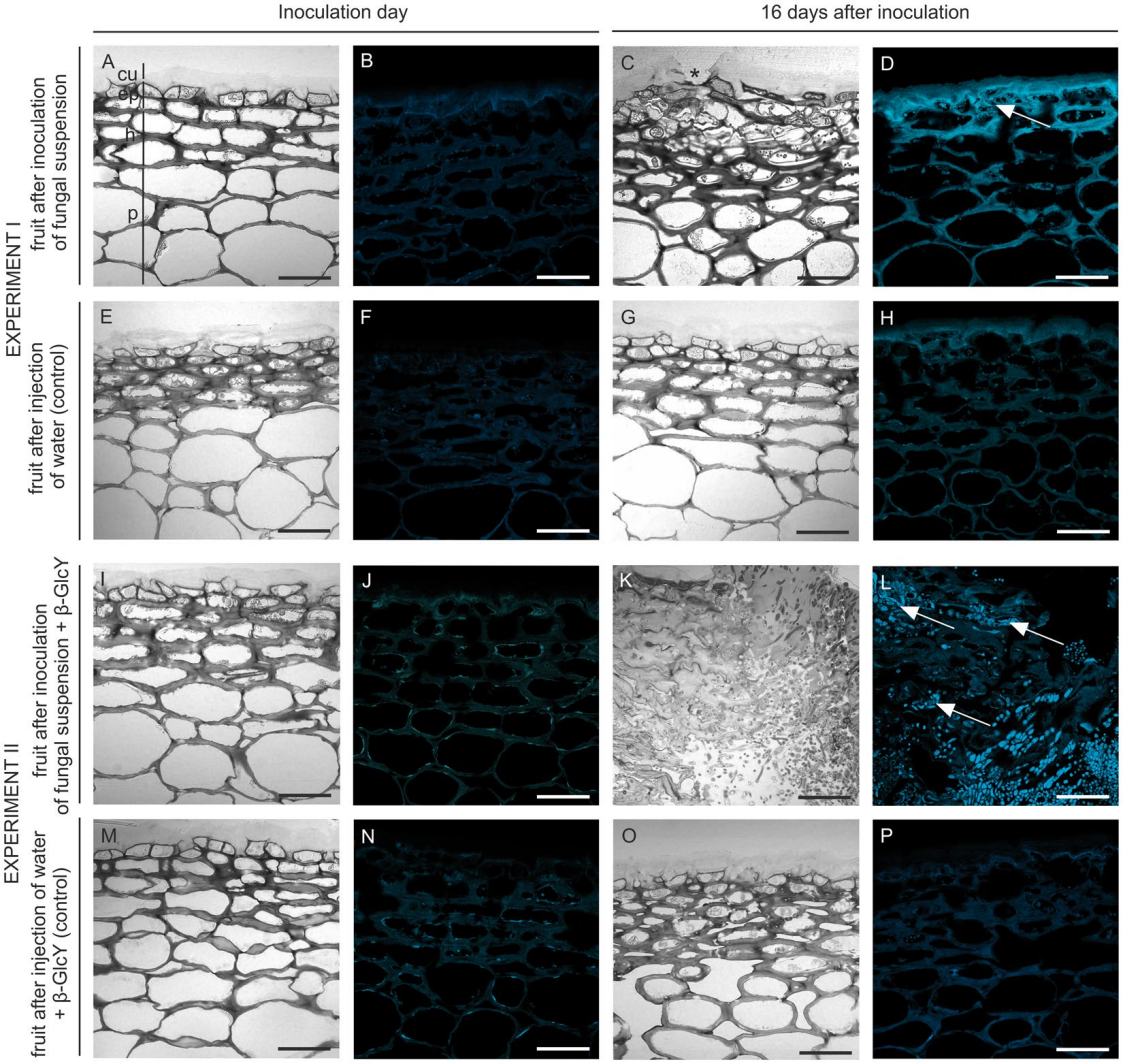
Comparative anatomies of the apple fruit tissue and callose presence at the beginning (**A**,**B**) and after 16 days of fungal infection (**C**,**D**). Images of equivalent control samples in experiment I (**E**–**H**). Sections of apple tissue after injection with addition of the β-GlcY on the onset day (I and J) and on day 16 of the experiment (**K**,**L**). Control samples in experiment II (M–P). Semi-thin sections stained with Toluidine blue (**A**,**E**,**I**,**M**,**C**,**G**,**K**,**O**) and with Aniline blue to detect callose (**B**,**F**,**J**,**N**,**D**,**H**,**L**,**P**). CLSM. Abbreviations: cu – cuticle; ep – epidermal layer; h – hypodermal layer; p – parenchyma. Bars 50 µm.

There were no histological changes, such as a different cell shape and cell layer numbers, on the first date of the experiment (Fig. 2A). Also, aniline blue staining showed the absence of callose on the inoculation day (Fig. 2B). In turn, variations in tissue morphology were observed in the apple fruits after 16 days of infection (Fig. 2C). The presence of fungal spores in the tissue since day 16 resulted in infection-associated modification of external epidermis and hypodermis. Moreover, the continuity of the cuticle covering the external epidermal layer was interrupted, which was visible as a microcracks (Fig. 2C, asterisked), also callose was accumulated in the disorganized exocarp (Fig. 2D, arrow). In control samples were no changes, and callose was not observed (Fig. 2E–H).

In experiment II, morphological changes (Fig. 2I) and callose secretion (Fig. 2J) were not observed on the inoculation day. The addition of β-GlcY to the fungal suspension caused complete destruction of the tissue, strong morphological alterations with absence of a remarkable border of the cells, and cytoplasm disorganization after 16 days of experiment (Fig. 2K). In fruit tissue with developing necrosis, there were intercellular spaces colonized by regular round fungal conidia (Fig. 2K). Staining of the samples from the β-GlcY-treated and fungus-infected fruits helped to detect the appearance of callose in the necrotized tissue close to *P. spinulosum* spores surrounded by cytoplasmic remnants (Fig. 2L, arrows). The control samples were characterized by the presence of small points with damaged adjacent cells from the external to internal layers as a result of water injection (Fig. 2M). In some cases, detachment of the apple skin from the parenchyma tissue was observed (Fig. 2O). Callose was not noticeable in the control samples (Fig. 2N,P).

### Qualitative analysis of the localization of AGPs epitopes in the fruit cell wall as a result of fungal infection

#### JIM13

**Figure 3:**
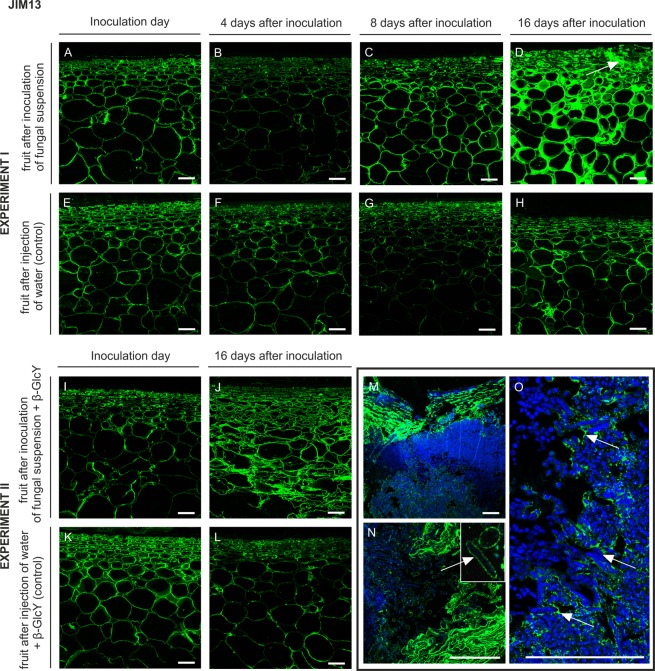
Changes in AGP immunolocalization recognized by the JIM13 antibody in apple tissue at different time points (0, 4, 8, 16 days) of infection by *P. spinulosum* (**A**–**D**) and in equivalent control samples (**E**–**H**). Immunolocalization of the JIM13 epitope in tissue treated with an inoculum with the β-GlcY on the inoculation day (**I**) and on day 16 after inoculation (**J**). Control samples (**K**,**L**). Magnification of infected parts of fruits after 16 days of experiment II (**M**–**O**). CLSM. Immunolabelling reaction with JIM13 mAb and Calcofluor White. Bars 100 µm.

The JIM13 antibody binds the β-GlcA-(1 → 3)-α-GalA-(1 → 2)-α-Rha motif of AGPs. In the section of the fruit tissue from the inoculation day (Fig. 3A), day 4 (Fig. 3B), and day 8 (Fig. 3C) after the inoculation, the JIM13 epitope was distributed throughout the cell wall, restricted to the wall region close to the plasma membrane. After 16 days of infection, the labelling was stronger and revealed the presence of AGPs on the whole surface of the cell wall, in cytoplasm compartments, and in intercellular spaces. Moreover, abundant JIM13 epitopes were detected in the disrupted spot of tissue external layers (Fig. 3D, arrow). In the control samples, the cell walls were highly labelled with JIM13 regardless of the time of the experiment. Binding of JIM13 to fungal-untreated sections of the apple tissue indicated the presence of AGP in the cells of all fruit layers, at the cell wall-plasma membrane border (Fig. 3E–H).

An unexpectedly high level of recognition of AGPs by JIM13 was observed in sections on day 16 after the fungal injection and treatment with β-GlcY (Fig. 3J), in comparison with the sections from the inoculation day (Fig. 3I) and the control samples (Fig. 3K,L). The higher magnification showed that binding was considerably increased by the substantial damage to the fruit tissue (Fig. 3M). The immunofluorescence labelling demonstrated the distribution of the AGP epitope on surfaces filled by fungal spores (Fig. 3O, arrows). Also, single *P. spinulosum* conidia were surrounded by AGPs (Fig. 3N, magnification).

#### LM2

**Figure 4:**
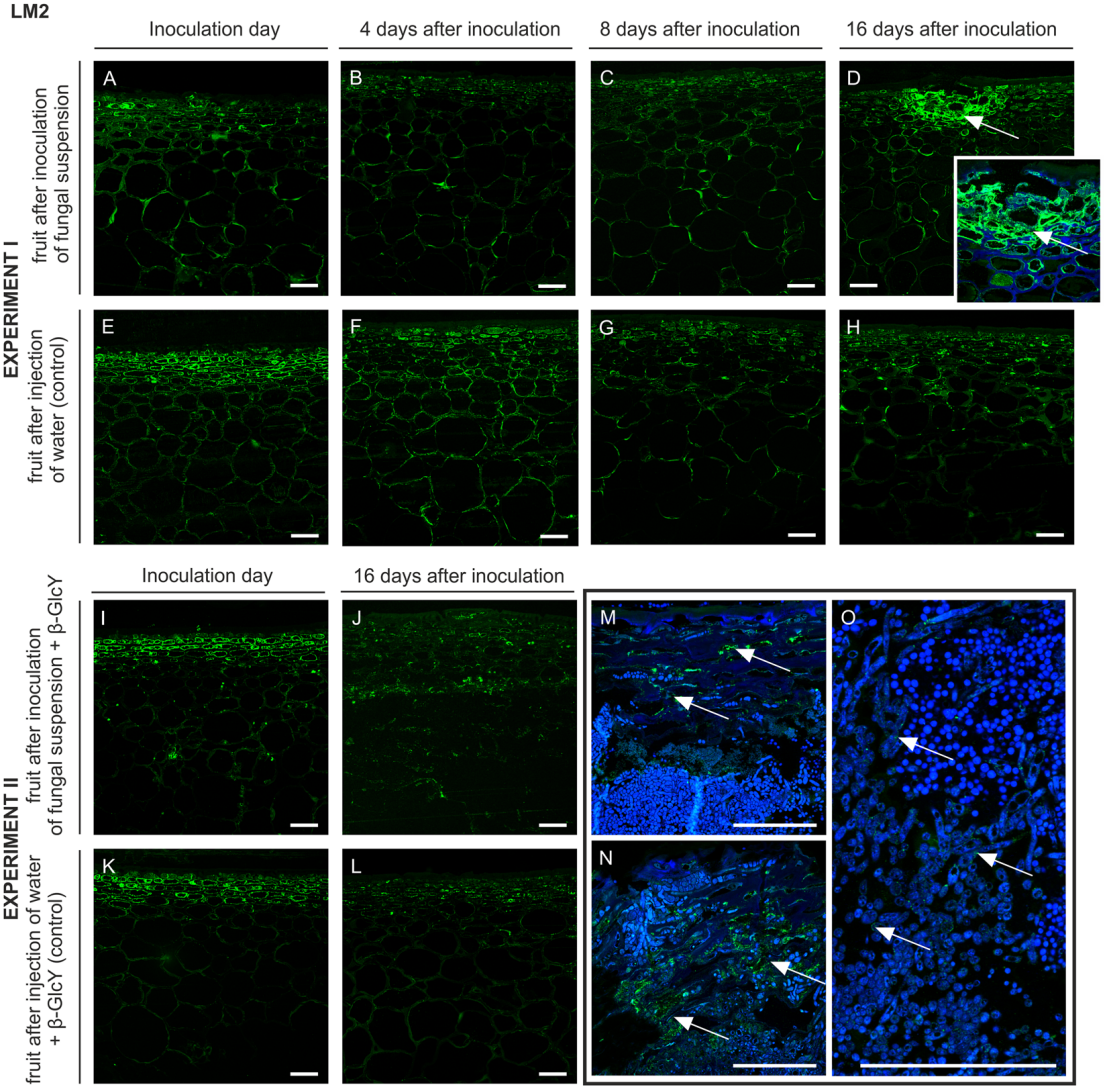
Changes in AGP immunolocalization recognized by the LM2 antibody in apple tissue at different time points (0, 4, 8, 16 days) of infection by *P. spinulosum* (**A**–**D**) and in equivalent control samples (**E**–**H**). Immunolocalization of the LM2 epitope in tissue treated by inoculum with the β-GlcY on the inoculation day (**I**) and on day 16 after the inoculation (**J**). Control samples (**K**,**L**). Magnification of infected parts of fruit after 16 days of experiment II (**M**–**O**). CLSM. Immunolabelling reaction with LM2 mAb and Calcofluor White. Bars 100 µm.

A similar pattern of labelling but a much weaker signal was revealed by the reaction with the LM2 antibody, which binds to (1 → 6)-β-Gal units with terminal β-GlcA in AGP chains. In the tissue after the inoculation of the fungal suspension, the arrangement of the LM2 epitopes was restricted to the cell wall-plasma membrane in the epidermis, hypodermis, and parenchyma at the inoculation day (Fig. 4A), day 4 (Fig. 4B), and day 8 (Fig. 4C) of the experiment. After 16 days of the fungal infection, the disrupted spots of the external layers were more abundant in the LM2 epitopes (Fig. 4D with magnification, arrows), in comparison to control samples (Fig. 4E–H).

At the beginning of experiment II, no difference in the fluorescence signal was revealed in fruit cells after inoculation of the fungal suspension with β-GlcY, in comparison to experiment I. AGP epitopes were visible mainly in the epidermal and hypodermal layers (Fig. 4I). Disorder in the LM2 epitope distribution was observed after 16 days (Fig. 4J). Mentioned changes were not observed in control samples (Fig. 4K,L). Higher magnification showed that in completely changed tissue (Fig. 4M, arrows) the fluorescence was limited to single points, i.e. randomly damaged surfaces filled by fungal spores (Fig. 4N,O, arrows).

#### LM14

**Figure 5:**
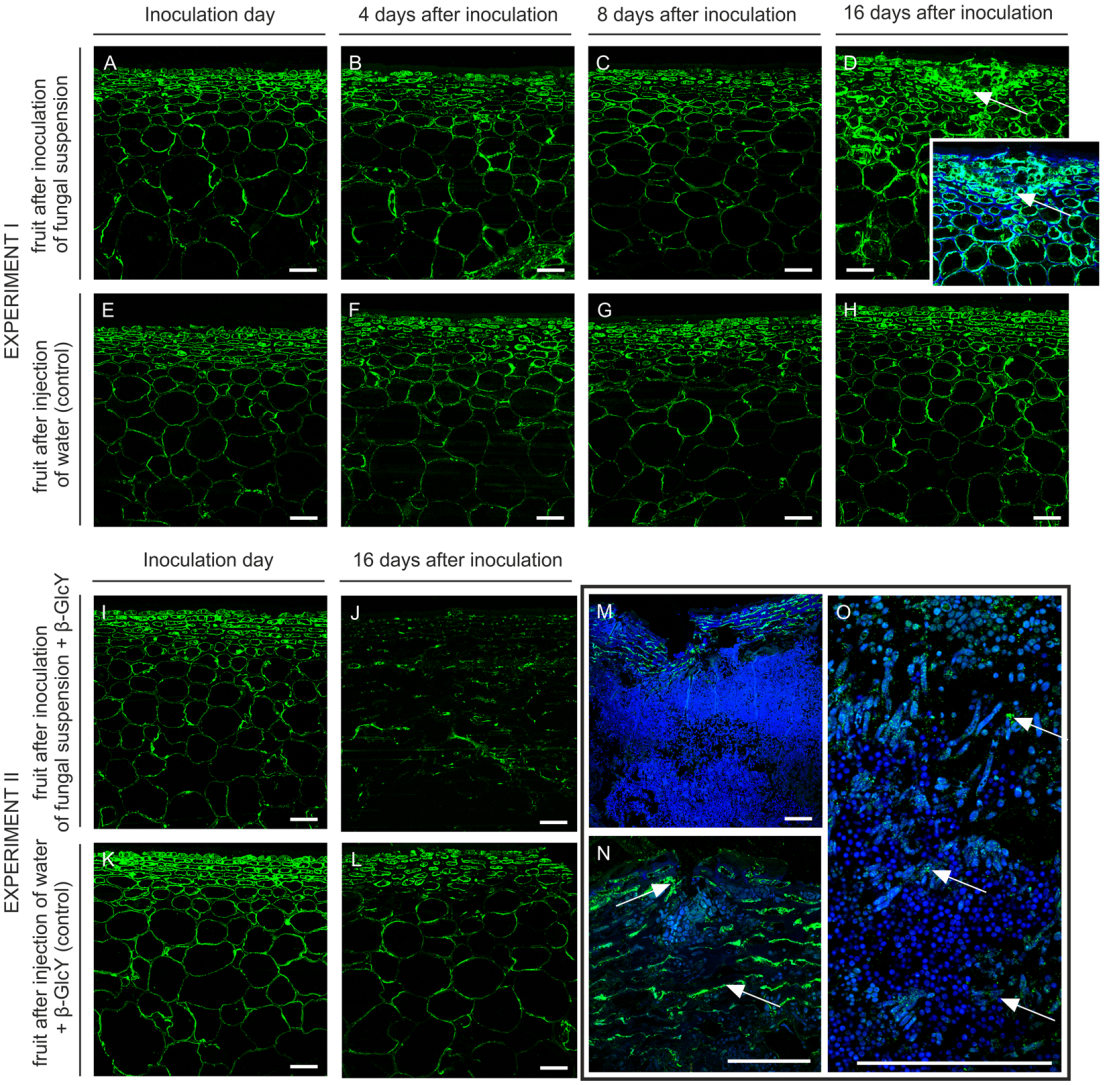
Changes in AGP immunolocalization recognized by the LM14 antibody in apple tissue at different time points (0, 4, 8, 16 days) of infection by *P. spinulosum* (**A**–**D**) and in equivalent control samples **(E**–**H**). Immunolocalization of the LM14 epitope in tissue treated by inoculum with the β-GlcY on the inoculation day (**I**) and on day 16 after inoculation (**J**). Control samples (**K**,**L**). Magnification of infected parts of fruit after 16 days of experiment II (**M**–**O**). CLSM. Immunolabelling reaction with LM14 mAb and Calcofluor White. Bars 100 µm.

The binding of the LM14 antibody recognizing arabinose- and galactose-enriched carbohydrate chains of AGPs is shown in Fig. 5. On day 4 (Fig. 5B) and 8 (Fig. 5C) after the inoculation, the LM14 epitope was present in the cell wall with an adjacent plasma membrane, similar to labelling on the inoculation day (Fig. 5A). Fungal infection lasting for 16 days influenced the spatial distribution of the LM14 epitope. In the destroyed layers of the apple with visible disruption of tissue continuity, a stronger fluorescence signal was observed in the cell walls of newly formed modifications (Fig. 5D with magnification, arrows). Mentioned spatio-temporal changes were not observed in control samples (Fig. 5E–H).

In experiment II, the spatial distribution of the LM14 epitope differed between the samples after fungal infection (Fig. 5I) and the control (Fig. 5K and L). After 16 days in tissue with significant infection progress, the LM14 antibody labelled damaged cell walls-plasma membrane (Fig. 5J). The fluorescence signal was dotted and much weaker than in control samples. In some fruits with accumulated *P. spinulosum* spores, the LM14 epitope was localised in damaged tissue (Fig. 5M,N, arrows) and close to conidia (Fig. 5O, arrows).

#### MAC207

**Figure 6:**
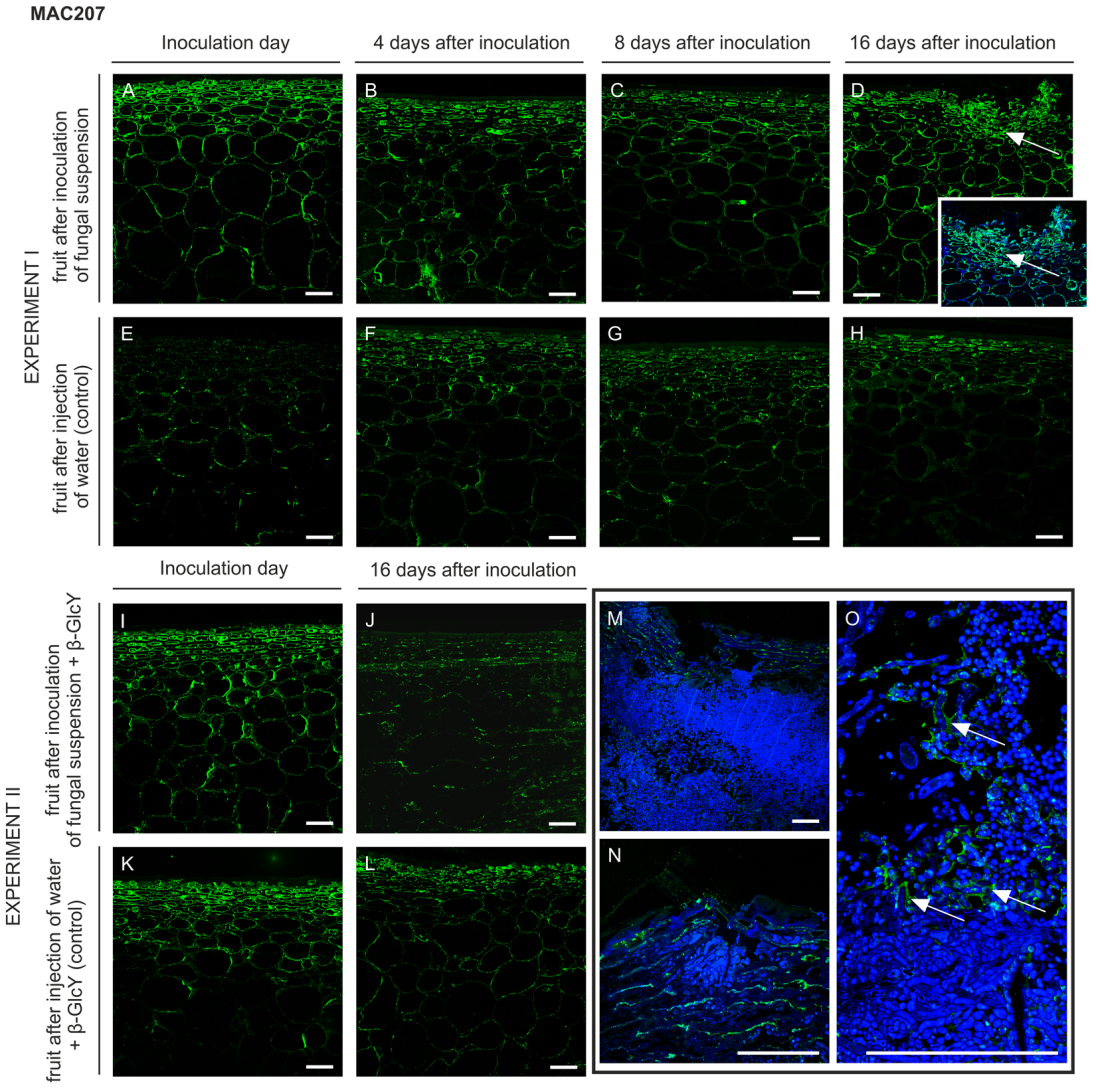
Changes in AGP immunolocalization recognized by the MAC207 antibody in apple tissue at different time points (0, 4, 8, 16 days) of infection by *P. spinulosum* (**A**–**D**) and in equivalent control samples (**E**–**H**). Immunolocalization of the MAC207 epitope in tissue treated by inoculum with the β-GlcY on the inoculation day (**I**) and on day 16 after inoculation (**J**). Control samples (**K**,**L**). Magnification of infected parts of fruit after 16 days of experiment II (**M**–**O**). CLSM. Immunolabelling reaction with MAC207 mAb and Calcofluor White. Bars 100 µm.

The MAC207 antibody recognises trisaccharides β-GlcA-(1 → 3)-α-GalA-(1 → 2)-α-Rha of glycan chains of AGPs. The pattern of labelling with MAC207 is similar to that observed after using the LM2 antibody. The labelling pattern was similar in the epidermis and hypodermis at the beginning of infection (Fig. 6A), and the spatial distribution was not changed during the progress of infection (Fig. 6B,C). However, after 16 days of the experiment, the localization of the MAC207 epitope was restricted to the site of injection in the external part of fruits (Fig. 6D with magnification, arrows). Lack of changes was noticed in fruit after injection of water in control samples (Fig. 6E–H).

In experimental and control samples, labelling was similar in the epidermis and hypodermis at the beginning of infection (Fig. 6I,K). On day 16 after the inoculation of the fungal suspension with β-GlcY, immunocytochemical analysis of the tissue showed a reduced fluorescence signal, also in the most damaged part of the tissue (Fig. 6J), in comparison to control sample (Fig. 6L). Higher magnification of these regions revealed disarrangement of the MAC207 epitope, which was noted in the surface filled by *P. spinulosum* conidia (Fig. 6M,N). Interestingly, the fluorescence was apparent as a single layer around the spores (Fig. 6O, arrows).

**Figure 7:**
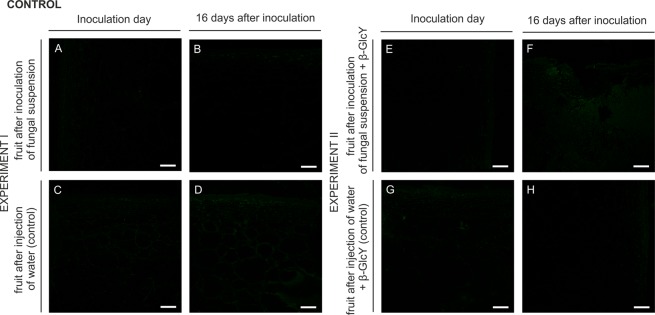
Control reactions for the immunofluorescence technique in experiment I (**A**–**D**) and II (**E**–**H**). CLSM. Bars 100 µm.

Since the primary antibodies were excluded from the immunolabelling procedure, no fluorescence was observed in both experiments, as shown in Fig. 7.

### Analysis of changes in the distribution of AGP epitopes in fruit during fungal infection

An overview of the immunofluorescence imaging of changes in the presence of AGPs in the infected fruits is summarized in Tables 1 and 2. The analysis of the spatial localization of the AGP epitopes revealed that the effect of the fungal infection on changes in the cell wall and the aforementioned modifications are observed at both the tissue and cellular levels, mainly on day 16 after inoculation. The pattern of labelling mainly with the JIM13 and LM14 antibodies was the most distinct on day 16 in comparison to the beginning of the experiment and the control samples. Disorder in the AGP distribution was visible in all parts of the fruits and the epitopes were detected throughout the epidermal layer to the parenchyma. The observation at the cellular level confirmed previous reports that AGPs occur in the cell wall-plasma membrane continuum. After 16 days of fungal infection, the AGP epitopes were present also in cytoplasm compartments in the infected fruit, indicating that the changes are spatially regulated within the extracellular matrix during the fruit disease.

**Table 1:** Analysis of the changes in spatial distribution of AGP epitopes, detected in fruit after fungal infection (inoculation day and 16th day of fungal inoculation).

Experiment I		Tissue level						Cellular level			
		Epidermal layer		Hypodermal layer		Parenchyma		Cell wall - plasma membrane		Cytoplasm compartments	
mAb		Treatment						Treatment			
		C	FI	C	FI	C	FI	C	FI	C	FI
Inoculation day in	JIM13	+	+	+	+	+	+	+	+	−	−
	LM2	+	−/+	+	−/+	−/+	−/+	+	+	−	−
	LM14	+	+	+	+	+	+	+	+	−	−
	MAC207	−	+	−	+	−/+	+	−/+	+	−	−
16 days 16	JIM13	+	++	+	++	+	++	+	++	−	++
	LM2	−/+	+	−/+	−/+	−/+	−/+	−/+	+	−	+
	LM14	+	++	+	++	+	++	+	++	−	+
	MAC207	+	+	−/+	+	−	+	−/+	+	−	+

Immunolabelling was evaluated according to Xie and co-workers (5): (−) no labelling, epitope not detected; (+) middle labelling; (++) very strong fluorescence signal. Abbreviations: C – control sample; FI – fruit after fungal infection

The addition of the β-GlcY caused progression of the infection and thus destruction of the apple tissue, which resulted in an unclear pattern of AGP labelling with all the antibodies used. Analysis at the tissue level showed disorderly changes in AGP localization after 16 days of the experiment over the entire fruit surface. The progress of the fungal disease was correlated with displacement of AGP and disturbance in their uninterrupted occurrence in the cell wall-plasma membrane continuum.

**Table 2:** Analysis of the changes in spatial distribution of AGP epitopes, detected in the fruit after fungal infection and β-GlcY treatment (inoculation day and 16th day of fungal infection).

Experiment II		Tissue level						Cellular level			
		Epidermal layer		Hypodermal layer		Parenchyma		Cell wall - plasma membrane		Cytoplasmic remnants	
mAb		Treatment						Treatment			
		C	FI	C	FI	C	FI	C	FI	C	FI
Inoculation day in	JIM13	+	+	+	+	+	+	+	+	−	−
	LM2	+	+	+	+	−/+	−/+	+	+	−	−
	LM14	++	++	++	++	+	+	+	+	−	−
	MAC207	+	+	+	+	−/+	−	+	+	−	−
16 days 16	JIM13	+	+	+	++	+	++	+	++	−	++
	LM2	−/+	−/+	−/+	−/+	−/+	−/+	−/+	−/+	−	+
	LM14	+	−/+	+	−/+	+	−/+	+	−/++	−	+
	MAC207	+	−/+	+	−/+	+	−/+	+	−/+	−	−

Immunolabelling was evaluated according to Xie and co-workers (5): (−) no labelling, epitope not detected; (+) middle labelling; (++) very strong fluorescence signal. Abbreviations: C – control sample; FI – fruit after fungal infection.

**Figure 8:**
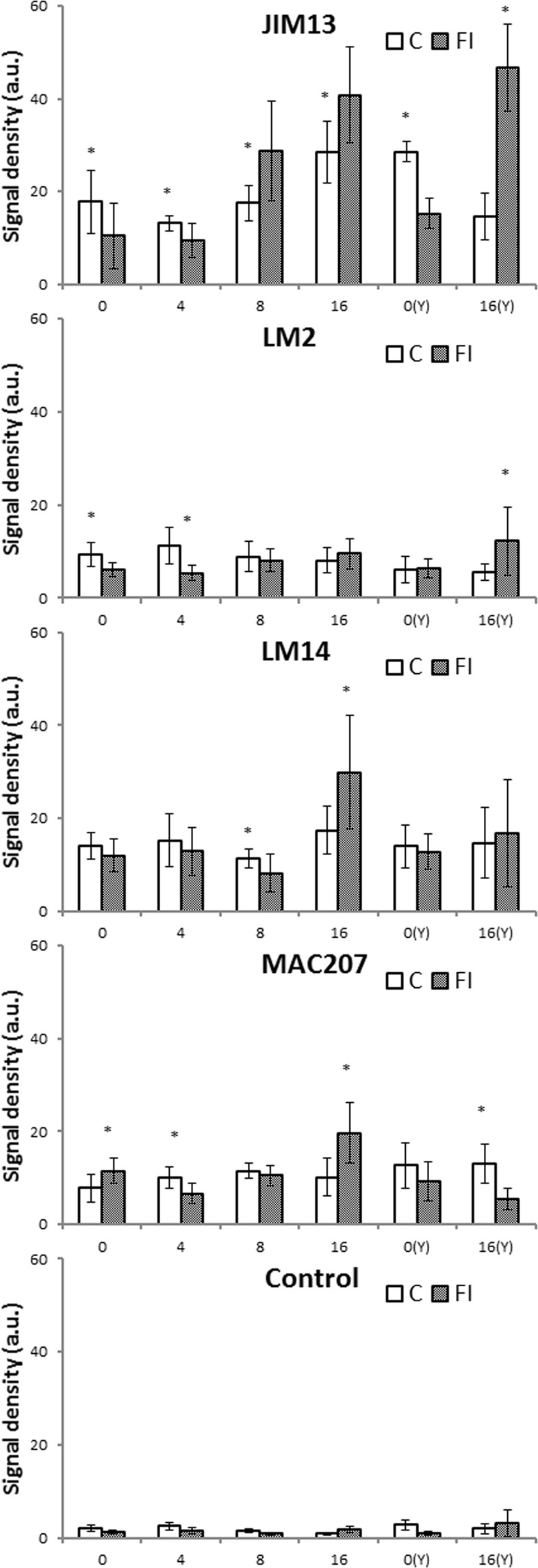
Quantification of immunofluorescence labelling. Density of the fluorescence signal of AGPs labelled by JIM13, LM2, LM14, and MAC207 antibodies and in the control reaction in apple fruit after fungal inoculation on days 0, 4, 8, 16 and after addition of the β-GlcY at 0 day – 0(Y) and after 16 days – 16(Y). Asterisks represent statistically significant differences (p < 0.05) between control (C) and sample after fungal infection (FI). Abbreviations: C – control sample; FI – fruit after fungal infection.

Analysis of quantification of the fluorescence signal density revealed differences in the abundance of the examined epitopes between the infected and control fruit. As shown in Fig. 8, the JIM13 antibody provided the most intensive signal in both experiments. Furthermore, signal density after labelling with JIM13 was most varied, and changed significantly in the experimental conditions. The abundance of JIM13 epitopes increased along with the progress of the fruit disease. Also, the β-GlcY treatment for 16 days resulted in a dramatic increase in the epitope abundance in the infected fruit. In the case of the LM14 and MAC207 antibodies, the signal density was relatively weak and the changes were smaller. However, after 16 days of the fungal injection, the epitopes became more abundant, which resulted in an increase in optical density compared to the control fruit. No obvious change was noticed in the signal intensity in the case of the LM2 antibody.

The differences between the antibodies used resulted from the pattern of distribution of the examined epitopes and their appearance in the most damaged fruit zones, while optical density of fluorescence was measured from all fruit layers from the epidermis to the parenchyma. Due to presence of the JIM13 epitopes in the whole fruit surface, the level of optical density of JIM13 fluorescence was much higher and similar to the description of the dynamic spatial presence in response to fungal disease.

## Discussion

Pathogens, including various *Penicillium* species, cause losses in fruit production. One of the most commonly reported postharvest disease of apple fruit is soft rot - blue mould. This highly damaging decay of apples is caused by *Penicillium* spp. characterized by high survivability due to their ability to produce numerous resilient conidia and proliferate rapidly on the tissue surface^31^. The experiments with the use of e.g. biospeckle imaging of infected fruits from the day of pathogen inoculation showed that development of necrosis and the onset of the defence mechanism occurred even on the first day of fungal disease. Biospeckle activity undergoes changes during fungal infection, and the initial visual signs of infection are related to suppression of the photosynthesis rate and cessation of this process at the later stages^32^. In our studies, the first macroscopically clear symptoms of disease appeared on day 4 of infection. Importantly, typical changes caused by blue mould such as massive colonization of macerated tissue by *P. spinulosum* were observed on day 16 after the infection took place. The morphological changes in the apple tissue were correlated with modifications in the AGP distribution. In this paper, detection of the spatio-temporal dynamics of the presence of AGPs in infection-associated alterations in the fruit cell wall was carried out. The visualization of the AGP epitopes by immunocytochemical imaging allowed determination of the changeable occurrence of AGPs in the fruit as a presumable response to fungal disease (experiment I) and definition of the effect of the β-GlcY-disrupting agent on the fruit tissue during fungal infection (experiment II).

Significant changes in the distribution of the examined cell wall components were observed on day 16 of the experiment in fruit with visual signs of the fungal disease. The earlier alterations in the AGP arrangement on days 4 and 8 after the inoculation were less pronounced. A stronger immunofluorescence signal was observed in samples with a higher level of cell degradation and disruption of primary cell walls, which indicates that progress in fungal infection is connected with a higher amount of AGPs. Interestingly, the AGP epitopes were more abundant in the damaged exocarp, and more precisely, in cells with marked cytoplasm disorganization. Immunofluorescence indicating the presence of the JIM13 and LM2 epitopes was evidently enhanced in the disrupted spots, in infection-associated modification of the cell wall during the following days of disease development.

An increase in the cell wall components as a signal of activation of defence responses and regulation of disease resistance is usually associated with cell wall-derived molecules, peptides^33^. Also increased secretion of callose in the amorphous matrix surrounding cytoplasmic remnants, in which *Penicillium digitatum* spores were trapped, was observed in infected citrus fruit. Accumulated callose deposits form an impervious composite with protective properties as a physical barrier against pathogen attack. Callose-enriched dome-shape parts are reinforcement of the cell wall at sites of attempted fungal penetration^3,34^. These observations are in agreement with our current studies, in which callose deposits were accumulated in particularly altered apple fruit tissue. Together, these results show that AGPs with other polysaccharides, such as callose, may form a barrier impermeable to fungal spores and influence the infection progress by surrounding the spores in infection sites. The role of AGPs was also proposed in the case microbial root infection, where AGPs were required for successful formation of infectious structures and modulation of plant immune response^8^.

The cell wall degradation process responsible for maceration of fruit tissue as a result of *Penicillium* spp. infection is caused by activation of e.g. pectinases inducing pectin depolymerization and thus their amount decreases^16^. Given the structure of AGPs and the presence AG chains attached to the protein core, it may be postulated that AGP glycan moieties also undergo enzymatic hydrolysis. However, an opposed symptom of fungal disease was observed in our studies and the examined AGPs were up-regulated in the infected fruits. According to earlier studies on tomato fruits, another explanation for the increased presence of AGPs may be their contribution to wound response. Molecular data obtained in tomato proved that the AGP gene expression level was induced within 1 h of pericarp excision^35^. In the case of the infected apples, there was a significant difference in the fluorescence signal between the damaged parts of the fruits and the control samples. The abundant presence of the AGP epitopes in the disorganized tissue, in sites highly modified by fungal spores, underlines their involvement in response to stress conditions.

AGPs act as modifiers of cell wall architecture through the cross linking of β-1,3-galactan chains with other cell wall polysaccharides. Thus, binding these moieties of AGPs by the β-GlcY allows analyses of the cell wall without normal specific connections^25^. In studies on suspension-cultured cells, AGP perturbations induced by β-GlcY treatment activated the intracellular programmed cell death (PCD) signal transduction pathway. Chaves and co-workers^36^ revealed that AGPs bound by the β-GlcY in the plasma membrane released intracellular Ca^2+^, which caused production of hydrogen peroxide and subsequent PCD. Another explanation for the aggregation of β-GlcY-AGP complexes in the plasma membrane-cell wall interface is disruption of linkages with cell components, triggering the PCD process^37^. Here, we report that application of the β-GlcY to infected apple fruits influences the rate of fungal disease. In turn, the rapid colonization of fungal spores in apple tissue is related to simultaneous alterations in the cell wall and an increase in the AGP distribution. After 16 days, there were markedly disrupted infection sites with aggregation of the cytoplasmic content in intercellular spaces, in comparison to infected samples without the β-GlcY addition. Labelling mainly with the JIM13 antibody showed that the amount of AGP epitopes increased in all the examined parts of tissue, and AGP epitopes appeared in aggregated remnants of necrotized tissue and precisely surrounded single fungal spores. Interestingly, the addition of the β-GlcY to the control apple fruit had no significant effect on the changes in apple tissue. These results indicate that the absence of proper cross-linking between AGPs and other cell wall constituents in stress conditions has an impact on the defence against fungal attack. Tissue damaged earlier under biotic stress undergoes more rapid processes leading to cell breakdown and cytoplasmic shrinkage, which underlines the importance of the cell wall in the resistance mechanism.

The molecular mechanisms of the AGP role in response to unfavourable environmental conditions are multifaceted, as AGP can be up-regulated and down-regulated. Under biotic stress, AGPs are involved in rigidification of the cell wall by oxidative cross-linking, release of free sugars protecting plant cells, stress signalling response, and formation of a protective buffer zone between membranes and cell wall matrix^38^. To sum up, fungal attack induces specific changes in the cell wall and up-regulation of AGPs in fruit tissue. Based on our findings, the explanation of this process may be the same as in the case of the root, where AGPs are constituents of biofilm representing a barrier that limits pathogen infection. The enhanced local accumulation of AGPs at infected sites plays protective functions during development of fruit disease and may be a part of the strategy of defence of apple fruits against biotic stress.

## Conclusion

1. Fungal infections influence the morphology and chemical composition of the fruit tissue. Degradation of the cell wall caused by *P. spinulosum* colonization and the progress of the infection process are associated with callose deposit accumulation and with changes in the presence of AGPs in fruit external layers and parenchyma.
2. The 16-day fungal disease results in the disruption of the specific pattern of AGP distribution and an increase in their presence in infection-associated modifications in fruit tissue.
3. Inactivation of AGP function by binding with the β-GlcY cause faster progress of fungal infection, which indicates that the absence of their normal action has an impact on the disorder in the cell wall-plasma membrane continuum.

### Highlights

- The presence of arabinogalactan proteins (AGPs) in apple fruit was visualized using the immunocytochemical technique.
- *Penicillium spinulosum* caused remarkable necrotic changes after 16 days of infection.
- Fungal infection induced changes in the presence of AGPs in fruit tissue.
- AGPs are distributed mainly in infection-associated modifications of fruit tissue.
- β-glucosyl Yariv reagent has an impact on the progress of fungal disease in fruit.
